# Schistosome egg antigen stimulates the secretion of miR-33-carrying extracellular vesicles from macrophages to promote hepatic stellate cell activation and liver fibrosis in schistosomiasis

**DOI:** 10.1371/journal.pntd.0011385

**Published:** 2023-05-30

**Authors:** Xin Qi, Yanan Pu, Fanyan Chen, Liyang Dong, Yongbin Ma, Junling Wang, Guo Yin, Di Lu, Xiaojun Chen, Jifeng Zhu, Yalin Li, Sha Zhou, Chuan Su

**Affiliations:** 1 Center for Global Health, Jiangsu Key Laboratory of Pathogen Biology, Department of Pathogen Biology and Immunology, Nanjing Medical University, Nanjing, Jiangsu, China; 2 Department of Outpatient & Emergency Management, the First Affiliated Hospital of Nanjing Medical University, Nanjing, Jiangsu, China; 3 Department of Nuclear Medicine, the Affiliated Hospital of Jiangsu University, Zhenjiang, Jiangsu, China; University of Manchester, UNITED KINGDOM

## Abstract

Schistosomiasis is a serious and neglected disease with a high prevalence in tropical and subtropical countries. The primary pathology of hepatic schistosomiasis caused by *Schistosoma japonicum* (*S*. *japonicum*) or *Schistosoma mansoni* (*S*. *mansoni*) infection is egg-induced granuloma and subsequent fibrosis in the liver. Activation of hepatic stellate cells (HSCs) is the central driver of liver fibrosis. Macrophages (Mφ), making up 30% of cells in hepatic granulomas, directly or indirectly regulate HSC activation by paracrine mechanisms, via secreting cytokines or chemokines. Currently, Mφ-derived extracellular vesicles (EVs) are broadly involved in cell communication with adjacent cell populations. However, whether Mφ-derived EVs could target neighboring HSCs to regulate their activation during schistosome infection remains largely unknown. Schistosome egg antigen (SEA) is considered to be the main pathogenic complex mixture involved in liver pathology. Here, we demonstrated that SEA induced Mφ to produce abundant extracellular vesicles, which directly activated HSCs by activating their autocrine TGF-β1 signaling. Mechanistically, EVs derived from SEA-stimulated Mφ contained increased miR-33, which were transferred into HSCs and subsequently upregulated autocrine TGF-β1 in HSCs through targeting and downregulating SOCS3 expression, thereby promoting HSC activation. Finally, we validated that EVs derived from SEA-stimulated Mφ utilized enclosed miR-33 to promote HSC activation and liver fibrosis in *S*. *japonicum*-infected mice. Overall, our study indicates that Mφ-derived EVs play important roles in the paracrine regulation of HSCs during the progression of hepatic schistosomiasis, representing a potential target for the prevention of liver fibrosis in hepatic schistosomiasis.

## Author summary

Schistosomiasis is a neglected parasitic disease affecting over 250 million people in tropical and subtropical regions. Among the common pathogenic species, *Schistosoma japonicum* and *Schistosoma mansoni* mainly cause hepatic schistosomiasis, which is mainly attributed to liver-trapped schistosome eggs, leading to granulomatous inflammation and liver fibrosis. HSC activation is the central driver of liver fibrosis during hepatic schistosomiasis. Macrophages (Mφ) are tightly connected to HSC activity. Currently, much evidence supports the pivotal role of Mφ-derived extracellular vesicles (EVs). However, the potential involvement of Mφ-derived EVs in HSC communication and modulation in hepatic schistosomiasis remains largely unknown. In this study, the authors illustrate that *S*. *japonicum* infection utilizes Mφ-derived EVs to promote HSC activation and liver fibrosis. Briefly, schistosome egg antigen (SEA) induces Mφ to secrete abundant miR-33-enriched EVs, which are transferred to enhance autocrine TGF-β1 production by targeting SOCS3 in HSCs, thereby promoting HSC activation and liver fibrosis. These discoveries provide a previously unrecognized Mφ-EVs-based therapeutic target regarding HSC activation, which can be manipulated to prevent liver fibrosis during hepatic schistosomiasis.

## Introduction

Schistosomiasis is a major neglected tropical disease caused by parasitic worms affecting approximately 250 million people worldwide. Among the species of the greatest clinical and socioeconomic importance causing human schistosomiasis, *Schistosoma japonicum* (*S*. *japonicum*) and *Schistosoma mansoni* (*S*. *mansoni*) cause hepatic schistosomiasis, which is one of the most prevalent chronic liver diseases. Much of the morbidity of hepatic schistosomiasis is attributed to schistosome eggs trapped in the liver, which release highly immunogenic and cytolytic egg antigens, inducing a granulomatous inflammation and thereby leading to granuloma formation and hepatic fibrosis [1–5].

Activation of quiescent hepatic stellate cells (HSC) into proliferative and fibrogenic myofibroblasts is well known as a central driver of liver fibrosis in both *S*. *japonicum* and *S*. *mansoni* infections, as well as other liver diseases [5,6]. Activated HSCs are the principal collagen-producing cells and are responsible for synthesizing abundant fibril-forming collagen (type I and III collagens) and extracellular matrix protein α-smooth muscle actin (α-SMA) in the fibrotic liver. HSC activation consists of two major phases, initiation, and perpetuation. Initiation is mainly dependent on paracrine signals from adjacent inflammatory cells, whereas perpetuation requires both paracrine and autocrine loops. Foremost among a number of secreted factors is transforming growth factor-β (TGF-β), which is well known as the most potent profibrotic cytokine to promote HSC activation, and also acts as a critical autocrine positive regulator to perpetuate HSC activation [7–9].

Liver macrophages (Mφ), derived from both resident Kupffer cells and circulating monocytes, are mostly located around areas of liver damage and fibrosis. During schistosome infection, Mφ make up 30% of cells in hepatic granulomas, and schistosome eggs induce alternatively Mφ, which directly or indirectly regulate neighboring HSC activation by paracrine mechanisms, via secreting various soluble factors (e.g. cytokines, chemokines, and growth factors), and ultimately cause liver fibrotic pathology [4–6,10,11]. Nevertheless, the mechanisms by which liver Mφ regulates HSC activation and hepatic fibrosis are quite complex.

Extracellular vesicles (EVs) are generated by all cells with an average size of ~100 nm in diameter. Currently, they have been well recognized as vital information carriers of biologically active molecules (e.g. nucleic acids, proteins, lipids, and metabolites) for intercellular communications among cell populations [12,13]. Under different physiological and pathological conditions, various Mφ phenotypes release EVs with different biological functions and properties that exert regulatory effects in recipient cells [13–15]. Upon schistosome infection, considering a high degree of heterogeneity and plasticity of liver Mφ, the numbers and/or bioactive contents of Mφ-derived EVs may be specifically altered. However, the potential contribution of Mφ-derived EVs to HSC communication and modulation in liver fibrosis during schistosome infection remains largely unknown.

In this study, we revealed that schistosome egg antigen (SEA) stimulated macrophages to generate abundant extracellular vesicles, which mechanistically utilized enclosed miR-33 to enhance autocrine TGF-β1 production of HSCs through repressing SOCS3 expression and thereby promote their activation and liver fibrosis in *S*. *japonicum*-infected mice. Our findings presented herein unravel a previously unrecognized exosome-based therapeutic target of anti-fibrosis therapy for hepatic schistosomiasis.

## Materials and methods

### Ethics statement

All animal experiments were performed according to the Regulations for the Administration of Affairs Concerning Experimental Animals. All procedures of animal experiments were approved by the Institutional Animal Care and Use Committee (IACUC) of Nanjing Medical University (Permit Number: IACUC-1811012). Maximum efforts were made to relieve suffering for all procedures. Maximum efforts were made to minimize suffering.

### Cell lines and cell cultures

The murine cell lines RAW264.7 (Mφ) and JS1 (HSC) were cultured in complete Dulbecco’s Modified Eagle’s Medium (DMEM; Gibco, Grand Island, NY) supplemented with 10% (v/v) heat-inactivated fetal bovine serum (FBS; Gibco), 2 mM L-glutamine (Gibco), and antibiotics (100 U/ml penicillin and 100 μg/ml streptomycin; Gibco) at 37°C with 5% CO_2_.

For the transwell co-culture, JS1 cells (1 × 10^5^ cells/well) were seeded into the bottom chambers of transwell (0.8 μm) of 24-well plates (Corning, NY, USA). RAW264.7 cells were untreated or pretreated with 40 μg/ml SEA and/or 10 μM GW4869 (an exosome secretion inhibitor) for 24 h and then seeded into the top chambers of the transwell. After being cocultured for 24 h, JS1 cells were collected for further analysis. Cultured cells were incubated with Fixable Viability Stain 620 (BD Bioscience, San Jose, CA) to determine viability. HSC proliferation was measured by the CCK-8 Cell Proliferation Detection Kit (KeyGEN Biotech, Nanjing, China).

### Egg isolation and antigen preparation

Schistosome eggs were isolated from the livers of *S*. *japonicum*-infected mice by sieving and enzymatic methods [16,17]. Briefly, the egg-trapped livers were chopped finely and homogenized in ice-cold PBS. The homogenate was sequentially filtered through 80-, 120-, and 160-mesh sieves to remove tissue clumps and debris. Eggs were recovered from the filtrate by passing over a 320-mesh sieve. The eggs on the screen were collected into a 50-ml tube and washed with PBS by low-speed (400g) centrifugation. The pellet was then resuspended in 50 ml PBS containing 10 mg collagenase B, 125 mg trypsin, 10 μg penicillin, and 20 μg streptomycin, and the mixture was incubated with gentle shaking at 37°C overnight. The eggs were then washed three times with PBS by centrifugation (1,500 rpm, 4°C, 5 min). The eggs were resuspended in 2 ml PBS and applied to the top of a Percoll column, prepared by mixing 2.4 ml Percoll with 9.6 ml of 0.25 M sucrose in a 15-ml tube. The tube was centrifuged at 800 g for 10 min. The egg pellet was resuspended in PBS and washed 3 times. *S*. *japonicum* egg antigen was prepared from isolated schistosome eggs as previously described [18,19]. Briefly, cleaned eggs were homogenized with PBS and sonicated 4 times on ice for 1 min each time. The supernatant with soluble egg antigen (SEA) was recovered by centrifugation and filter-sterilized with a 0.22 μm filter (Merck Millipore, Darmstadt, Germany). Protein concentration was determined using a bicinchoninic acid protein assay kit (Thermo Scientific, Waltham, MA).

### Quantitative real-time PCR (qRT-PCR)

Total RNA was extracted from Mφ-EVs, SEA-Mφ-EVs, or cells using the MirVana RNA isolation kit (Ambion, Austin, TX) according to the manufacturer’s protocol. qRT-PCR primers were purchased from GeneCopoeia (Guangzhou, China) and listed in supplementary S1 Table. qRT-PCR reactions were performed using the All-in-one SYBR Green qPCR Mix (GeneCopoeia) on a CFX96 Real-Time PCR Detection system (Bio-Rad, Hercules, CA). The relative expression of mRNA or miRNA was calculated by the 2^(-ΔΔCt)^ method and normalized to the expression of β-actin or U48, respectively.

### Western blot analysis

The cells were lysed with RIPA lysis buffer (Beyotime, China). Protein extracts were separated by 12% sodium dodecyl sulfate-polyacrylamide gel electrophoresis (SDS-PAGE) and transferred to polyvinylidene difluoride membranes (Bio-Rad). The membranes were blocked with 5% milk in Tris-buffered saline containing 0.1% Tween-20 for 1 h at room temperature and then incubated with primary antibodies overnight at 4°C. After washing, the membranes were incubated with the horseradish peroxidase-conjugated secondary antibodies for 1h at room temperature. The enhanced chemiluminescence reagent (Thermo Fisher Scientific, Rockford, IL) and a ChemiDoc MP Imaging System (Bio-Rad) were used to visualize the immunoblots. GAPDH or β-actin was used as a protein loading control. The intensity of the protein bands was quantified using ImageJ software (NIH, Bethesda, MD; http://imagej.nih.gov/ij). Protein expression levels were normalized to the levels of GAPDH or CD63. The antibodies to CD63, calreticulin, TSG101, α-SMA, TGF-β1, SOCS3, SMAD3, p-SMAD3, GAPDH, β-actin, and HRP-linked anti-rabbit or anti-mouse IgG were purchased from Abcam (Cambridge, UK). The antibodies to collagen types I and III were purchased from Proteintech (Chicago, USA). The concentrations used for all the antibodies were listed in supplementary S2 Table.

### Enrichment, characterization, and RNase A of EVs from Mφ

RAW264.7 Mφ, cultured in DMEM (Gibco) containing 10% EV-depleted FBS, were stimulated with or without SEA (40 μg/ml) for 24 h, and then the supernatants were collected from cultures using centrifugation. Mφ-derived EVs were enriched from the supernatants as previously described with minor modifications [20]. Briefly, the supernatants were centrifuged at 2,000 × g for 20 min and filtered through a 0.45-μm filter (Merck Millipore) to remove cell debris. EVs were pelleted at 100,000 × g for 90 min and washed with PBS 2 times and finally were resuspended in PBS. Transmission electron microscopy, nanoparticle tracking analysis, and western blot analysis were used to confirm the presence and characteristics of enriched EVs. Briefly, transmission electron microscope analysis was used to confirm the enrichment of typical EVs with typical cup-like morphology. Nanoparticle tracking analysis using a NanoSight NS300 (Malvern Instruments Ltd., Worcestershire, UK) to determine the size distribution (with diameters ranging from 80 to 180 nm) and the number of enriched EVs. Western blot analysis was used to confirm the expression of common exosome markers (CD63, TSG101) in enriched EVs.

SEA-Mφ-EVs were treated with 2 μg/ml RNase A and 0.1% Triton X-100 for 20 min remove RNAs, which was then confirmed by agarose gel electrophoresis.

### Transmission electron microscopy (TEM) of EVs

The purified EVs were fixed with 4% paraformaldehyde (PFA) and 4% glutaraldehyde in phosphate buffer (pH 7.4) at room temperature. Then, the fixed EVs were applied to the carbon-coated copper mesh and soaked in a 2% phosphotungstic acid solution (pH 7.0) for 30 s and the mesh was inspected with a transmission electron microscope (JEM-1200EX; JEOL, Tokyo, Japan).

### EV labeling and cellular uptake

JS1 cells were labeled with 3,3’-dioctadecyloxacarbocyanine (DiO; green) at 37°C for 20 min and then washed twice with PBS. EVs enriched from the supernatants of RAW264.7 cells treated with or without SEA (40 μg/ml) were labeled with the membrane dye (CM-Dil; red) according to the manufacturer’s protocol. Labeled EVs were further pelleted by centrifuging at 100,000 × g for 1 h and washed twice with PBS. Following labeling, JS1 cells were incubated with fluorescently labeled EVs (0.75×10^9^ particles/ml) for 2 h and then fixed with 4% PFA. After fixation, cells were washed with PBS-0.5% Triton X-100 for permeability and then stained with 4,6-diamino-2-phenylindole (DAPI) to label the nucleus. All reagents were from Invitrogen (Carlsbad, California). A Nikon Eclipse Ti confocal laser scanning microscope (Nikon, Japan) was used to obtain images.

### Cell transfection

The synthetic miR-33 mimic, miR-33 inhibitor, mimic control, and inhibitor control were obtained from RiboBio (Guangzhou, China). The SOCS3 overexpression plasmid and matched control vector were purchased from GeneCopoeia. The JS1 cells or RAW264.7 cells at 60% confluency were transfected with miR-33 mimic/inhibitor (50 nM), SOCS3 plasmid (1 μg), or their matched controls using Lipofectamine 2000 (Invitrogen) in Opti-MEM medium (Invitrogen) according to the manufacturer’s procedures.

To prepare SEA-Mφ-EVs with miR-33 knockdown, RAW264.7 cells were transfected with unlabeled or Texas-Red-labeled miR-33 mimic (50 nM) using Lipofectamine 2000 in Opti-MEM medium and then stimulated with SEA (40 μg/ml) for 24 h after 48 h of transfection. Mφ-derived EVs with or without miR-33 knockdown were enriched from the supernatants as described above.

### Dual-luciferase assay

JS1 cells were co-transfected with 500 ng pmiR-Rb-report-SOCS3-3’-UTR (wild-type or mutant) and 100 nM miR-33 mimic/inhibitor or negative control (Biological Nuclear). After 48 h, the cells were lysed and luciferase activity was assessed with the LUC-Pair Duo-Luciferase Assay Kit 2.0 (GeneCopoeia) according to the manufacturer’s instructions.

### Mice

Eight-week-old male BABL/c mice were purchased from the Laboratory Animal Center of Nanjing Medical University (Nanjing, China). All mice were maintained in a specific pathogen-free breeding facility with strictly controlled humidity, temperature, and 12-h light/dark cycles.

### *S*. *japonicum* infection and animal treatment

Mice were percutaneously infected with 14 *S*. *japonicum* cercariae of the Chinese mainland strain from infected snails (*Oncomelania hupensis*) acquired from obtained from the National Institute of Parasitic Diseases, Chinese Center for Disease Control and Prevention in Shanghai.

*S*. *japonicum*-infected mice were randomly divided into four groups, 4 mice for each group. After 4 weeks post-infection, mice were injected with Mφ-EVs or SEA-Mφ-EVs (2×10^9^ in 200 μl PBS) via the tail-vein once a week for 4 consecutive weeks. SEA-Mφ-EVs with miR-33-knockdown were enriched from the supernatants of SEA-stimulated Mφ pre-transfected with a miR-33 inhibitor. Mice were sacrificed for further analysis one week after the last injection.

### Liver egg burden assessment

Briefly, the large left lobes of mouse livers were removed. A small piece of each tissue was cut out for pathological evaluation and the remaining tissue (~0.5 g) was weighed and stored at -80°C. Then the frozen tissues were digested with 5% KOH at 37°C overnight, and then the number of eggs per gram of liver tissue was determined by microscopic examination.

### Isolation of hepatic stellate cells

Primary HSCs were isolated from mouse livers as described previously [21]. Briefly, the livers in situ were perfused with an EGTA (ethylene glycol tetraacetic acid) solution (NaCl, 8 g/L; KCl, 0.4 g/L; NaH_2_PO_4_·H_2_O, 88.17 mg/L; Na_2_HPO_4_, 120.45 mg/L; HEPES, 2.38 g/L; NaHCO_3_, 0.35 g/L; EGTA, 0.19 g/L; Glucose, 0.9 g/L; pH, 7.4), followed by perfusion with an EGTA solution with 0.05% pronase (Roche, Rotkreuz, Switzerland) and 0.04% type IV collagenase (Invitrogen). The whole liver was then homogenized and digested with an EGTA solution containing 0.05% pronase, 10 U/ml DNase I (Sigma-Aldrich, St. Louis, MO), and 0.08% type IV collagenase for 30 min at 37°C. A 10% Optiprep (Axis-Shield, Oslo, Norway) solution was used for density gradient centrifugation to isolate HSCs. Finally, CD45^+^ cells were depleted from cell samples using CD45 magnetic beads (Miltenyi Biotech, San Diego, CA).

### Isolation of hepatic Mφ

Hepatic Mφ were isolated as described previously [22]. The liver single-cell suspension was prepared from fresh liver tissue and then filtered through a 200-mesh sieve. Hepatocytes were removed by two low-speed centrifugations (50g for 5 min). Liver mononuclear cells were separated by density centrifugation on a 25–50% Percoll gradient and then centrifuged at 800 × g for 30 min. Cells were collected from the layer between 25–50% gradient interface and then allowed to adhere for 3 h and washed to remove non-adherent cells.

### Liver histopathology and fibrosis measurement

Liver tissues were fixed in 4% buffered formalin, embedded in paraffin, and then sectioned (5~7 μm). Liver sections were stained with Masson’s trichrome for visualizing fibrosis. All the images were captured by using an Axiovert 200M microscope (Zeiss, Oberkochen, Germany) coupled with a digital camera.

Three discontinuous liver sections were evaluated for each mouse. Six to eight fields from each slide were randomly obtained and the blue-stained area per total area was determined with Image-Pro Plus 6.0 software (Media Cybernetics, Rockville, MD) for semi-quantitative analysis of hepatic fibrosis.

For detecting the liver collagen content, hydroxyproline content was measured using a Hydroxyproline assay kit (Nanjing Jiancheng Bioengineering Institute, Nanjing, China) as described by the manufacturer.

### Statistical analysis

The statistical analyses were performed with GraphPad Prism 5.0 (GraphPad Software Inc., La Jolla, CA). Data were expressed as mean ± SD. For comparing two and multiple continuous variables, Student’s *t*-test and one-way analysis of variance were used, respectively. *P*<0.05 was considered significant. The numerical data used in all figures are included in S1 Data.

## Results

### Inhibition of EVs secretion impairs the ability of SEA-stimulated Mφ to activate HSCs

**Fig 1:**
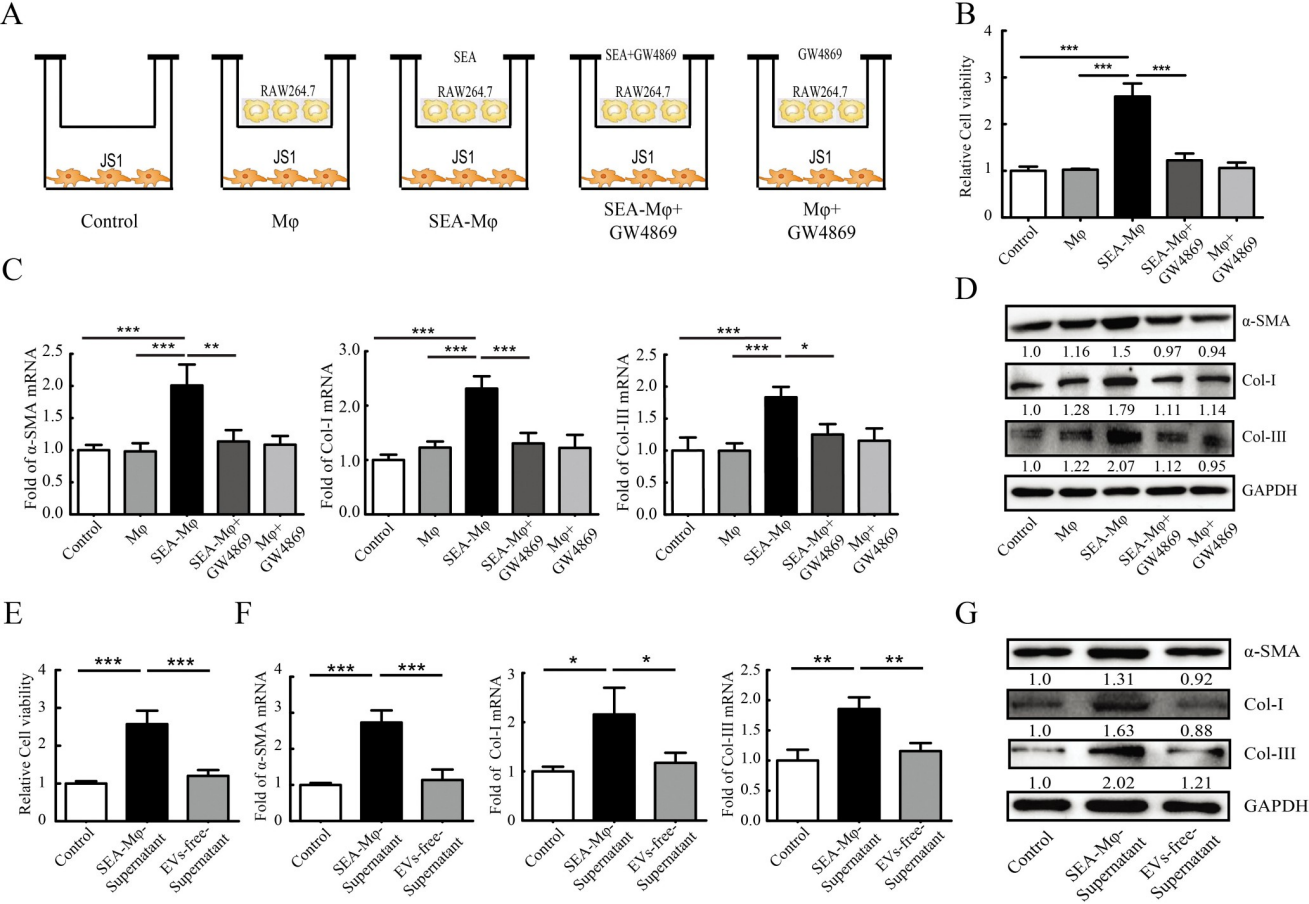
Inhibition of EV secretion impairs the ability of SEA-stimulated Mφ to activate HSCs. (A) Schematic diagram of the transwell co-culture system. Top chambers: RAW264.7 cells. Bottom chambers: JS1 cells. (B) The viability of JS1 cells. (C and D) qRT-PCR (C) and immunoblot analysis (D) of fibrotic markers (α-SMA, Col-I, and Col-III) in JS1 cells. (E-G) JS1 cells were cultured with complete supernatants or EVs-free supernatants from SEA-Mφ mono-cultures. The cell viability was determined (E). qRT-PCR (F) and immunoblot analysis (G) of fibrotic markers (α-SMA, Col-I, and Col-III) in JS1 cells. Numbers below the blots represent the quantification of the band intensities expressed as fold change over the control (same for all the immunoblots). All graph data are expressed as the mean ± SD of 3 biological replicates per group. *P < 0.05, **P < 0.01, ***P<0.001.

Mφ play an important role in regulating HSC activation in hepatic schistosomiasis [10]. We first used a transwell coculture system to investigate whether secreted factors derived from SEA-stimulated Mφ (SEA-Mφ) are involved in HSC activation (Fig 1A). Following coculture, HSCs cocultured with SEA-Mφ exhibited increased viability/proliferation, while pretreatment of SEA-Mφ with GW4869 (an exosome secretion inhibitor) prior to coculture, significantly inhibited HSC proliferation activity (Fig 1B). qRT-PCR and immunoblot analysis further revealed that coculture with SEA-Mφ significantly increased the mRNA and protein levels of fibrotic markers α-SMA, types I and III collagens (Col-I and Col-III) in HSCs. Consistently, GW4869 pretreatment of SEA-Mφ also markedly inhibited the expression of these fibrotic markers in HSCs of the coculture (Fig 1C and 1D). In addition, we further collected the supernatants from SEA-Mφ cultures and removed the EVs to further investigate their contribution to HSC activation. We observed that supernatants from SEA-Mφ cultures containing EVs significantly induced the activation of HSCs showing increased viability (Fig 1E) and the mRNA and protein levels of fibrotic markers (Fig 1F and 1G), but not EVs-free supernatants from SEA-Mφ cultures (Fig 1E–1G). These data suggest that EVs derived from SEA-stimulated Mφ make an important contribution to HSC activation.

### Mφ produce abundant extracellular vesicles upon SEA stimulation

**Fig 2:**
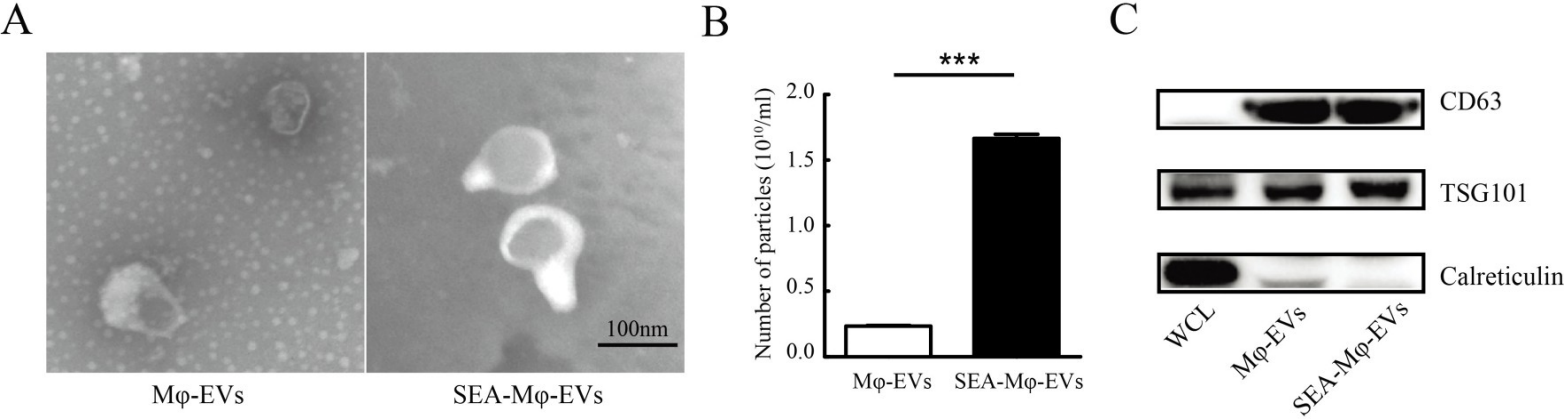
Mφ produce abundant extracellular vesicles upon SEA stimulation. (A) Representative transmission electron microscopy (TEM) images of Mφ-EVs and SEA-Mφ-EVs. Scale bar, 100 nm. (B) Nanoparticle tracking analysis (NTA) of Mφ-EVs and SEA-Mφ-EVs was performed to determine the counts (n = 3). (C) Western blot analysis of indicated exosome markers (positive markers: CD63 and TSG101; negative marker: calnexin) in whole cell lysates (WCL), Mφ-EVs, and SEA-Mφ-EVs. All graph data are expressed as the mean ± SD of 3 biological replicates per group. ***P<0.001.

We next characterize EVs derived from unstimulated-Mφ or SEA-Mφ (abbreviated as Mφ-EVs and SEA-Mφ-EVs, respectively). As shown in Fig 2A, transmission electron microscope (TEM) analysis confirmed the enrichment of typical EVs with a typical cup-like morphology in both groups. Simultaneously, no typical EVs were enriched from SEA, whereas numerous EVs were enriched from SEA-stimulated Mφ (S1 Fig). Nanoparticle tracking analysis (NTA) showed no significant difference in size between SEA-Mφ-EVs and Mφ-EVs, with diameters ranging from 80 to 180 nm (S2A and S2B Fig). Notably, SEA stimulated a 10-fold higher secretion of EVs from RAW264.7 macrophages compared to unstimulated control cells (Fig 2B). Additionally, immunoblot analysis revealed an enrichment of common exosome markers (CD63 and TSG101) in both Mφ-EVs and SEA-Mφ-EVs, along with trace expression of the organelle marker calreticulin, in comparison to respective whole cell lysates (WCL) (Fig 2C). These data demonstrate that SEA induces macrophages to produce abundant extracellular vesicles.

### EVs derived from SEA-stimulated Mφ directly activate HSCs

**Fig 3:**
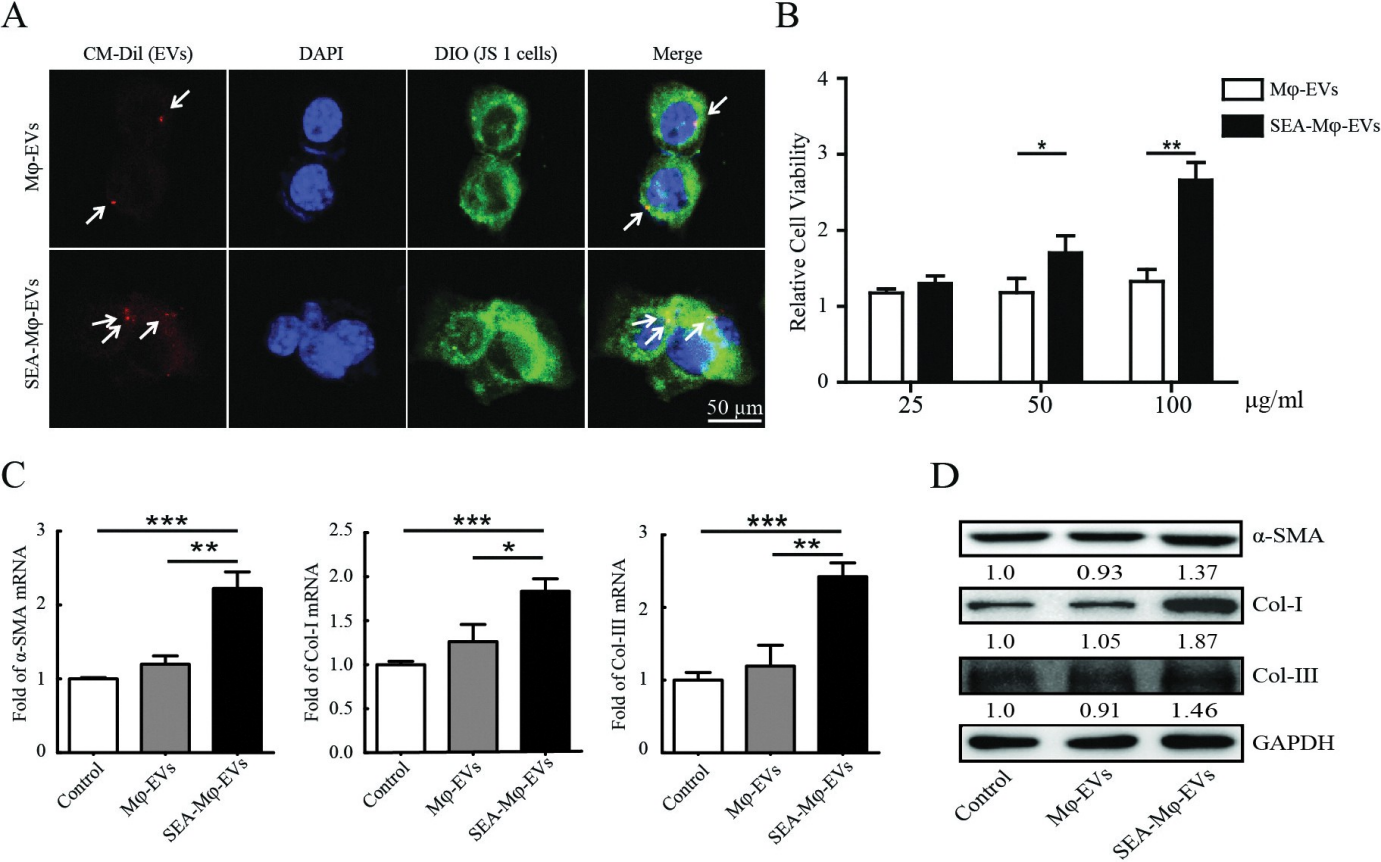
EVs derived from SEA-stimulated Mφ directly activate HSCs. (A) Confocal microscopy image of Dil-labelled EVs (red) incubated with JS1 cells. DAPI (blue) was used to stain nuclei and DIO (green) to label JS1 cells. Scale bar, 50 μm. (B) JS1 cells were treated with Mφ-EVs or SEA-Mφ-EVs (25, 50, 100 μg/ml) for 24 hours, and the cell viability was determined. (C and D) JS1 cells were treated with Mφ-EVs or S-Mφ-EVs (100 μg/ml). qRT-PCR (C) and immunoblot analysis (D) of fibrotic markers (α-SMA, Col-I, and Col-III) in JS1 cells were performed. All graph data are expressed as the mean ± SD of 3 biological replicates per group. *P < 0.05, **P < 0.01, ***P<0.001.

We next investigated whether SEA-Mφ-derived EVs have a direct effect on the activation of HSCs. Firstly, EVs were purified from Mφ with or without SEA treatment and labeled with a red fluorescent dye (Dil). Following treatment with Dil-labeled EVs, the bio-uptake of EVs in JS1 cells labeled with the lipophilic dye DiO (green) was verified by confocal microscopy (Fig 3A). We also observed that both Mφ-EVs and SEA-Mφ-EVs were located on the inner surface of the cell membrane or around the nuclei (Fig 3A), suggesting the uptake of EVs in JS1 cells through endocytosis. Subsequently, *in vitro* treatment with different concentrations of SEA-Mφ-EVs resulted in significantly increased viability of JS1 cells in a dose-dependent manner, whereas Mφ-EVs were unable to stimulate the activation of JS1 cells (Fig 3B). The 100 μg/ml EV concentration was selected for subsequent studies. In addition, qRT-PCR and immunoblot analysis further revealed that *in vitro* treatment with SEA-Mφ-EVs significantly increased the mRNA and protein levels of fibrotic markers α-SMA, Col-I, and Col-III in JS1 cells, whereas no alteration was observed with treatment with Mφ-EVs (Fig 3C and 3D). These results indicate that the EVs derived from SEA-stimulated Mφ directly activate HSCs.

### SEA-Mφ-EVs stimulate autocrine TGF-β1 signaling to activate HSCs

**Fig 4:**
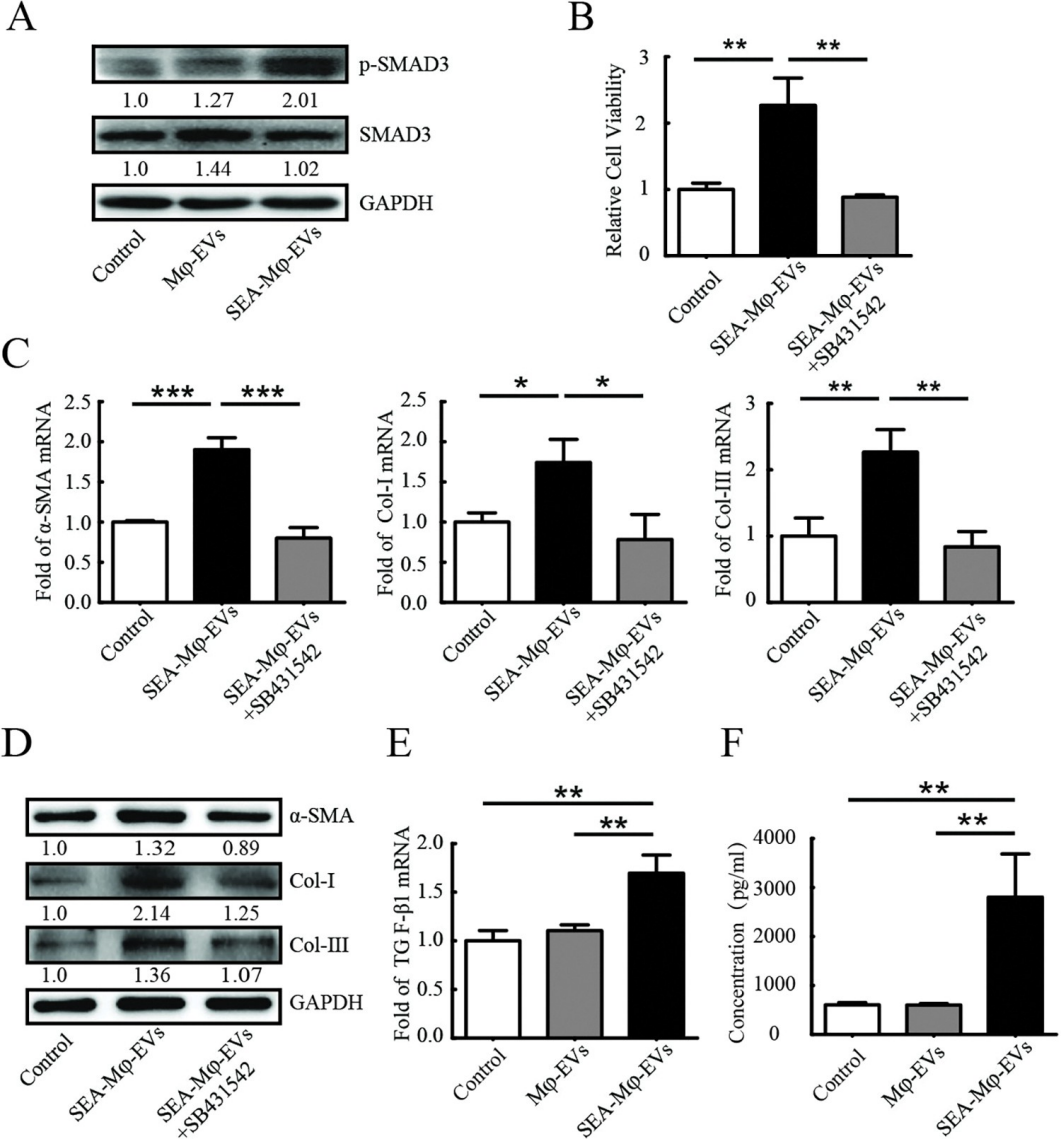
SEA-Mφ-EVs stimulate autocrine TGF-β1 signaling to activate HSCs. (A-D) JS1 cells were treated with Mφ-EVs or SEA-Mφ-EVs (100 μg/ml). Immunoblot analysis of p-SMAD3 and total SMAD3 in JS1 cells was performed (A). The cell viability was determined. The TGF-β type I receptor inhibitor SB431542 was used at 10 μM (B). qRT-PCR (C) and immunoblot analysis (D) of fibrotic markers (α-SMA, Col-I, and Col-III) in JS1 cells were performed. (E) JS1 cells were treated with Mφ-EVs or S-Mφ-EVs (100 μg/ml). qRT-PCR of TGF-β1 mRNA expression in JS1 cells was performed. (F) Supernatants were assayed for secreted TGF-β1 by ELISA. All graph data are expressed as the mean ± SD of 3 biological replicates per group. *P < 0.05, **P < 0.01, ***P<0.001.

Transforming growth factor-β (TGF-β) is generally considered the most potent cytokine to activate HSCs. TGF-β signals phosphorylate downstream SMAD proteins, predominantly SMAD3, which translocate into the nucleus to promote type I and III collagen transcription in HSCs [23]. We further investigated whether TGF-β-SMAD3 signaling is involved in SEA-Mφ-EVs-mediated HSC activation. As expected, we observed a significantly increased phosphorylation of SMAD3 (p-SMAD3) in JS1 cells with the treatment of SEA-Mφ-EVs (Fig 4A). Treatment with the TGF-β type I receptor inhibitor SB431542 almost completely abolished the ability of SEA-Mφ-EVs to increase the viability of JS1 cells (Fig 4B), and the ability to increase the mRNA and protein levels of fibrotic markers (α-SMA, Col-I, and Col-III) in JS1 cells (Fig 4C and 4D).

We next determined whether SEA-Mφ-EVs carry and directly transport TGF-β1 protein or mRNA to JS1 cells. However, western blot and PCR analysis showed a minimal and unchanged amount of TGF-β1 protein or mRNA in SEA-Mφ-EVs, the same as in Mφ-EVs (S3A and S3B Fig), suggesting that SEA-Mφ-EVs do not carry TGF-β1 protein or mRNA by themselves.

Autocrine TGF-β1 signaling acts as a positive regulator for enhancing or maintaining HSC activation [23]. Thus, we next investigated whether SEA-Mφ-EV-derived fractions promote autocrine TGF-β1 signaling in HSCs. As expected, following treatment with SEA-Mφ-EVs, we observed an increased mRNA expression of TGF-β1 in HSCs (Fig 4E). Further, ELISA was performed to confirm the increased autocrine production of TGF-β1 in the supernatants of JS1 cell culture (Fig 4F). Taken together, these results demonstrated that SEA-Mφ-EVs stimulate autocrine TGF-β1 signaling to activate HSCs.

### SEA-Mφ-EVs mediate the activation of HSCs via RNAs

**Fig 5:**
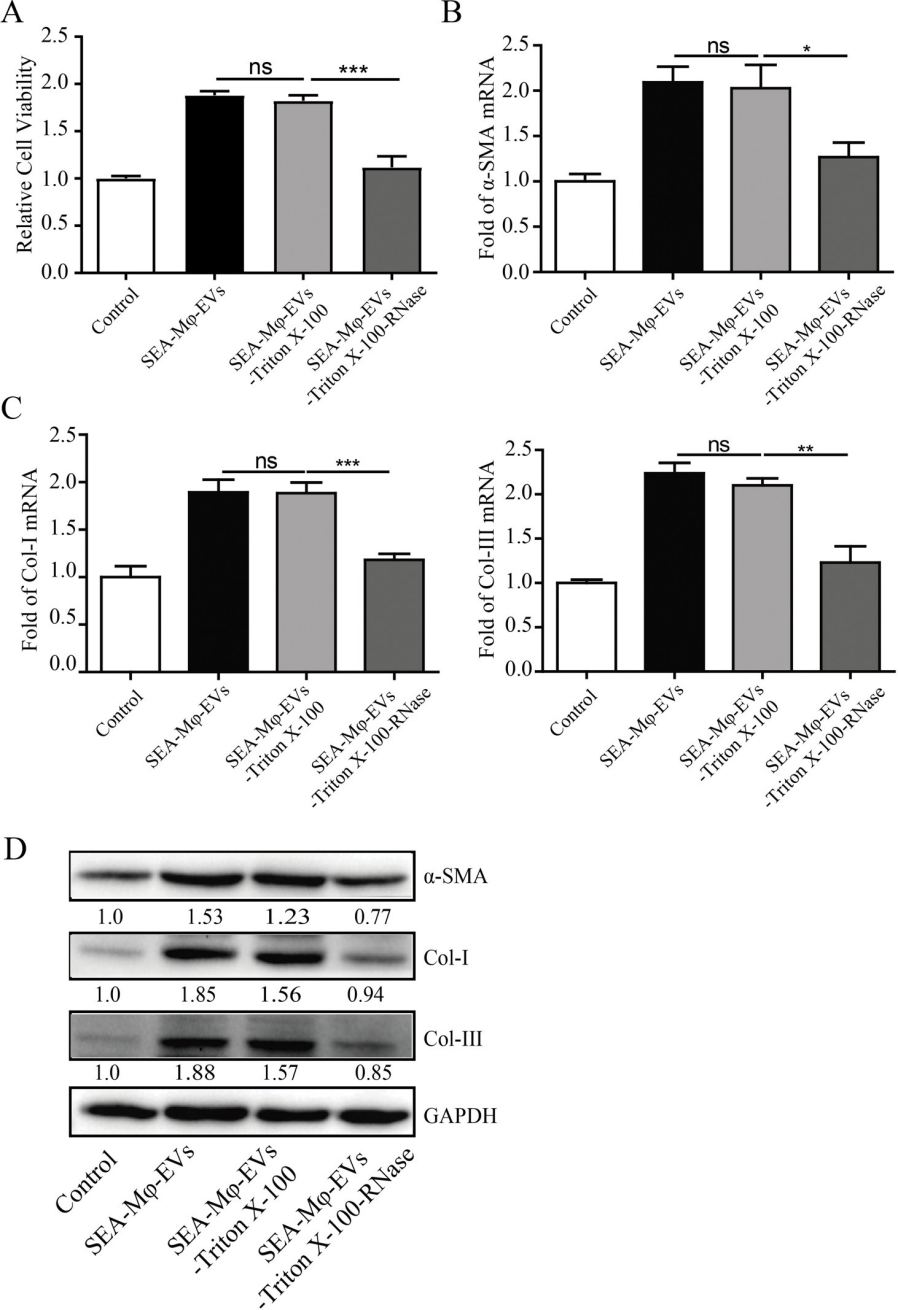
SEA-Mφ-EVs mediate the activation of HSCs via RNAs. (A) JS1 cells were incubated with SEA-Mφ-EVs with or without the removal of RNAs. The cell viability was determined. (B and C) qRT-PCR (B) and immunoblot analysis (C) of fibrotic markers (α-SMA, Col-I, and Col-III) in JS1 cells were performed. All graph data are expressed as the mean ± SD of 3 biological replicates per group. *P < 0.05, **P < 0.01, ***P<0.001.

We next explored the underlying mechanisms by which SEA-Mφ-EVs treatment induces autocrine TGF-β1 in HSCs. EVs are carriers enclosing various biomolecules to facilitate cross-talk between cells, predominantly rich in certain subsets of small non-coding RNAs and proteins [13,24]. RNAs were removed from SEA-Mφ-EVs, which was then confirmed by agarose gel electrophoresis (S4 Fig). Intriguingly, RNA removal largely impaired the ability of SEA-Mφ-EVs to activate JS1 cells (Fig 5A) and to increase the mRNA and protein levels of fibrotic markers (α-SMA, Col-I, and Col-III) (Fig 5B and 5C). These results indicated that RNAs in EVs are responsible for SEA-Mφ-EVs-mediated HSC activation.

### SEA-Mφ-EVs contain increased miR-33 which promotes HSC activation through the down-regulation of SOCS3

**Fig 6:**
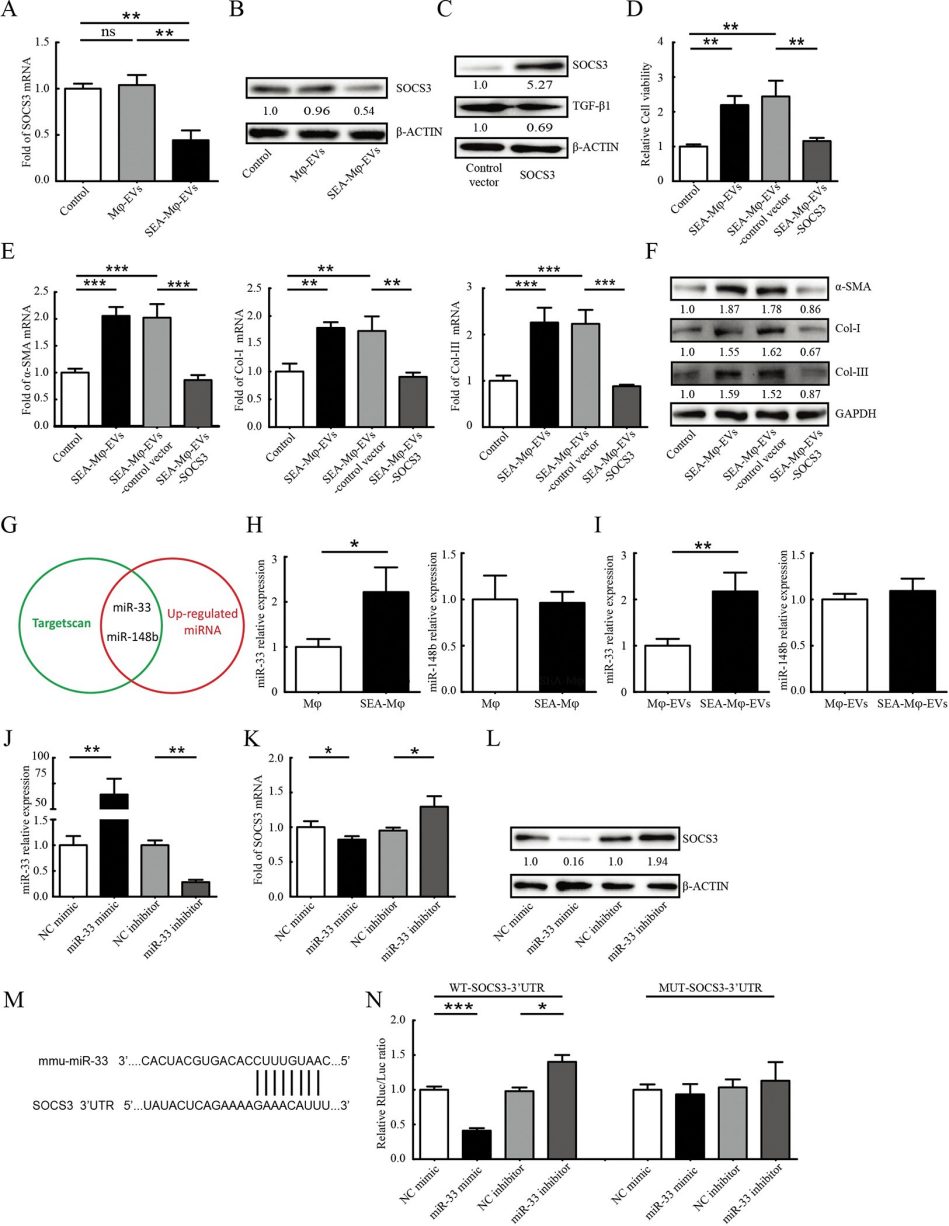
SEA-Mφ-EVs contain increased miR-33 which promotes HSC activation through the regulation of SOCS3. (A and B) JS1 cells were treated with SEA-Mφ-EVs (100 μg/ml). qRT-PCR (A) and immunoblot (B) analysis of SOCS3 in JS1 cells were performed. (C-F) JS1 cells were transfected with *SOCS3* overexpression vector or control-empty vector and then treated with SEA-Mφ-EVs (100 μg/ml). Immunoblot analysis of SOCS3 and TGF-β1 expression in JS1 cells is shown (C). The cell viability was determined (D). qRT-PCR (E) and immunoblot analysis (F) of fibrotic markers (α-SMA, Col-I, and Col-III) in JS1 cells were performed. (G) Venn diagrams showing overlapping miRNAs (miR-33 and miR-148b) between TargetScan predicted miRNAs targeting SOCS3 and the up-regulated miRNAs in the liver of *S*. *japonicum*-infected mice. (H) Relative expression levels of miR-33 and miR-148b in unstimulated-Mφ and SEA-Mφ. (I) Relative expression levels of miR-33 and miR-148b in EVs derived from unstimulated-Mφ and SEA-Mφ. (J-L) JS1 cells were transfected with mimic negative control or miR-33 mimic; inhibitor negative control or miR-33 inhibitor. Relative expression levels of miR-33 in JS1 cells were detected using qRT-PCR (J). qRT-PCR (K) and immunoblot (L) analysis of SOCS3 in JS1 cells were performed. (M) Schematic diagram showing the putative miR-33-binding site in the SOCS3 3’-UTR. (N) Effects of miR-33 on the activity of the luciferase reporter construct containing wild-type or mutant miR-33 binding site of the SOCS3 3’-UTR in JS1 cells transfected with miR-33 mimic or inhibitor. All graph data are expressed as the mean ± SD of 3 biological replicates per group. *P < 0.05, **P < 0.01, ***P<0.001.

EVs typically packed certain repertoires of microRNAs (miRNAs), silencing genes at the post-transcriptional level via transcript degradation in recipient cells [25,26]. The suppressor of the cytokine signaling-3 (SOCS3) has been reported to negatively regulate TGF-β1 production and liver fibrosis [27,28]. We next observed downregulated SOCS3 mRNA and protein levels in JS1 cells treated with SEA-Mφ-EVs (Fig 6A and 6B). The involvement of SOCS3 in regulating TGF-β1 expression was further investigated by overexpression experiments. We found that SOCS3-overexpressing JS1 cells expressed a lower level of TGF-β1 (Fig 6C). Importantly, the abilities of SEA-Mφ-EVs to increase the viability of JS1 cells (Fig 6D) and to increase the mRNA and protein levels of fibrotic markers (α-SMA, Col-I, and Col-III) in JS1 cells (Fig 6E and 6F) were essentially abolished by SOCS3-overexpression. The results suggest that SEA-Mφ-EVs-enclosed RNAs may promote HSC activation through down-regulating SOCS3 in HSCs.

We next explored SOCS3-targeted miRNAs. We then generated Venn diagrams to identify overlapping miRNAs between TargetScan-predicted miRNAs targeting SOCS3 and the up-regulated miRNAs in the liver of *S*. *japonicum*-infected mice [29] and identified miRNA-33 (miR-33) and miRNA-148b (miR-148b) as potentially targeting SOCS3 (Fig 6G). However, we observed only a significant up-regulation of miR-33 but not miR-148b in SEA-Mφ (Fig 6H) and SEA-Mφ-EVs (Fig 6I).

To further investigate whether miR-33 targets and regulates SOCS3 expression, we transfected JS1 cells with miR-33 mimic or inhibitor, which was shown to effectively increase or decrease miR-33 level, respectively (Fig 6J). In parallel, JS1 cells transfected with miR-33 mimic or inhibitor had decreased and increased SOCS3 expression, respectively, as determined by qRT-PCR and immunoblot (Fig 6K and 6L). To further investigate whether miR-33 directly binds the 3’-UTR of SOCS3 mRNA, a putative binding site of miR-33 was predicted by TargetScan (Fig 6M), and the wild-type 3’-UTR sequence (wt-SOCS3-3’-UTR) and the mutant 3’-UTR sequence (mut-SOCS3-3’-UTR) were cloned into luciferase reporter vectors, respectively. The luciferase reporter assay showed that miR-33 mimic decreased and miR-33 inhibitor increased the luciferase activity with the wt-SOCS3-3’-UTR but not with the mut-SOCS3-3’-UTR (Fig 6N).

Collectively, these results suggest that increased miR-33 enclosed in SEA-Mφ-EVs promotes HSC activation through the down-regulation of SOCS3.

### SEA-Mφ-EVs transfer miR-33 into HSCs and promoted their activation

**Fig 7:**
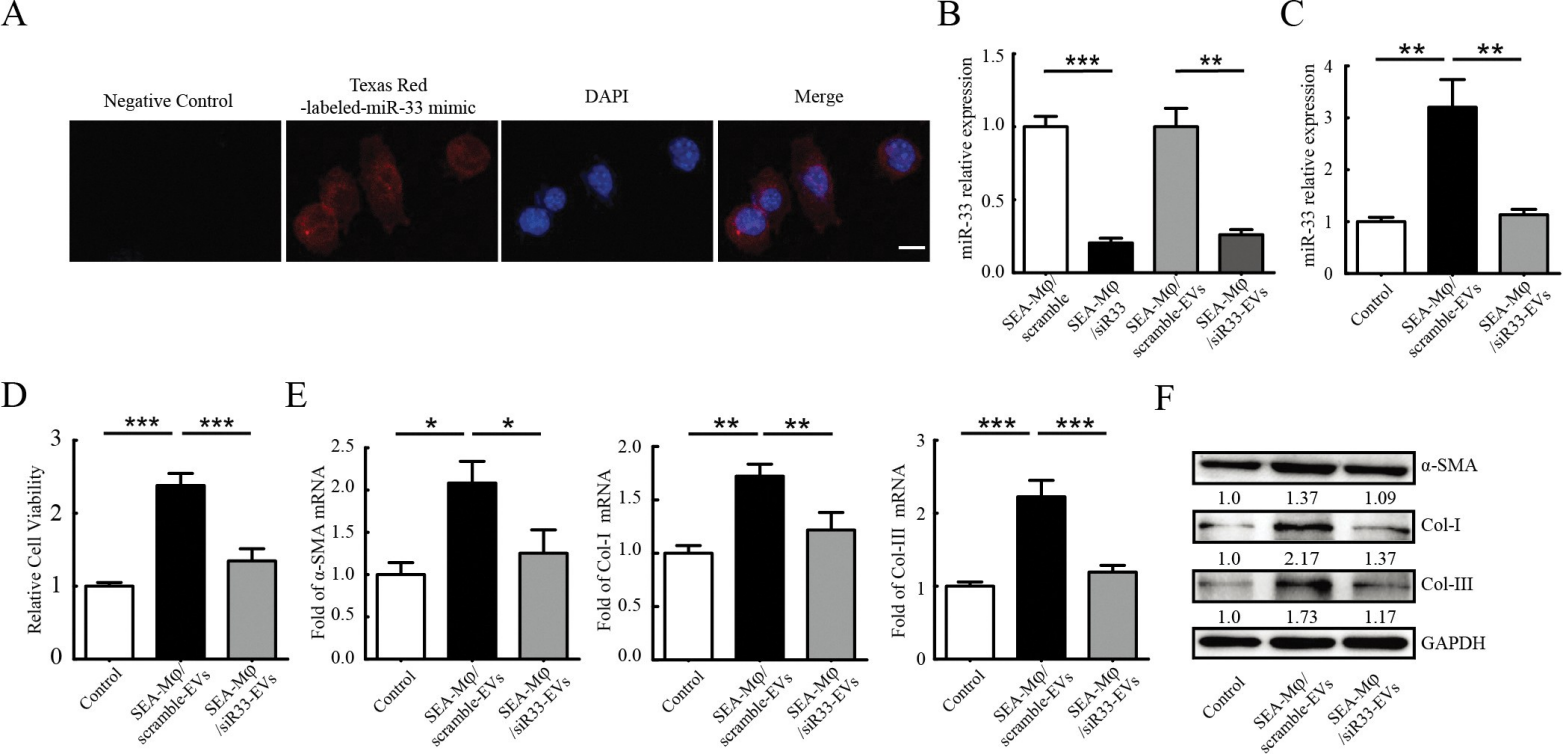
SEA-Mφ-EVs transfer miR-33 into HSCs and promoted their activation. (A) Confocal microscopy image showing JS1 cells incubated with EVs from SEA-Mφ pre-transfected with Texas Red-labeled-miR-33 mimic. DAPI (blue) was used to stain the nuclei of the JS1 cells. Scale bar, 50 μm. (B) Mφ were transinfected with miR-33 inhibitor and then stimulated with SEA (40 μg/ml) for 24 h. Relative expression levels of miR-33 in SEA-Mφ or SEA-Mφ-EVs were detected by qRT-PCR. (C-F) JS1 cells were treated with SEA-Mφ-EVs (100 μg/ml) with or without miR-33-knockdown for 24 hours. Relative expression levels of miR-33 in JS1 cells were determined using qRT-PCR (C). The cell viability was determined (D). qRT-PCR (E) and immunoblot analysis (F) of fibrotic markers (α-SMA, Col-I, and Col-III) in JS1 cells were performed. All graph data are expressed as the mean ± SD of 3 biological replicates per group. *P < 0.05, **P < 0.01, ***P<0.001.

We next investigated whether miR-33 transferred in SEA-Mφ-EVs are important for HSC activation. First, we assessed if SEA-Mφ-EVs could transfer miR-33 into HSCs. Following incubation with EVs derived from SEA-Mφ pre-transfected with Texas Red-labeled-miR-33 mimic, confocal fluorescence microscopy images revealed miR-33 distribution in the cytoplasm of JS1 cells (Fig 7A). We then used a miR-33 inhibitor to knock down the miR-33 level in SEA-Mφ, which resulted in a significant reduction of miR-33 level in SEA-Mφ, as well as in these SEA-Mφ-derived EVs (Fig 7B). As expected, JS1 cells treated with EVs from miR-33-knockdown SEA-Mφ also exhibited a significant reduction of miR-33 level (Fig 7C), suggesting that SEA-Mφ-EVs are responsible for the increased miR-33 expression in the recipient JS1 cells by transferring miR-33. Furthermore, miR-33-knockdown in SEA-Mφ largely impaired the ability of SEA-Mφ-EVs to activate JS1 cells (Fig 7D) and to increase the mRNA and protein levels of fibrotic markers (α-SMA, Col-I, and Col-III) (Fig 7E and 7F). Taken together, these results demonstrate that SEA-Mφ-EVs transfer miR-33 into HSCs and promote their activation.

### SEA-Mφ-EVs transfer miR-33 to promote HSC activation and liver fibrosis in *S*. *japonicum*-infected mice

**Fig 8:**
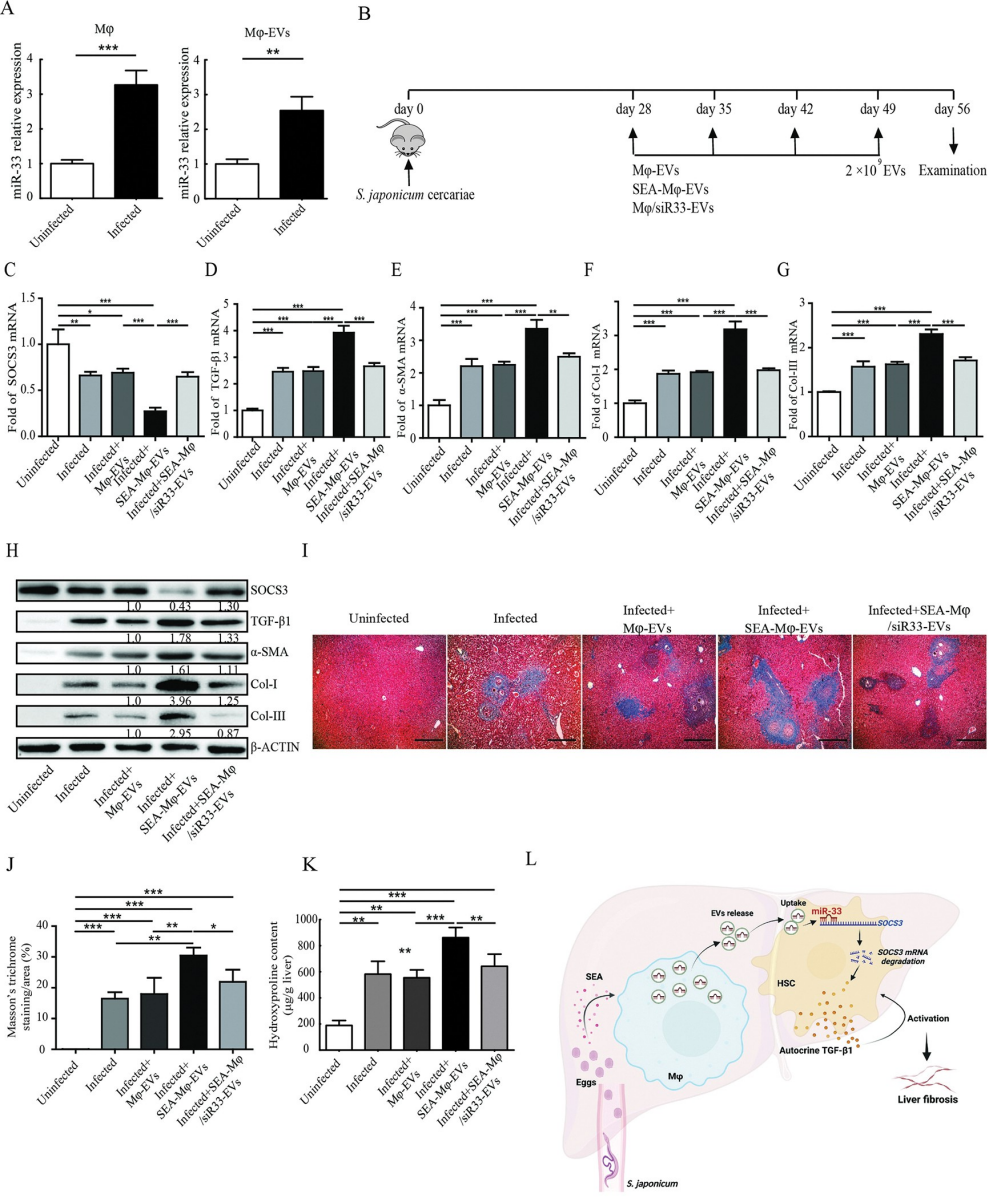
SEA-Mφ-EVs utilize enclosed miR-33 to promote HSC activation and liver fibrosis in *S*. *japonicum*-infected mice. (A) Relative expression levels of miR-33 in primary liver Mφ (left) or their bearing EVs (right) from normal mice or *S*. *japonicum*-infected mice (8 weeks post-infection) were measured using qRT-PCR. (B-K) Animal experimental design (B). *S*. *japonicum*-infected mice were injected with Mφ-EVs, SEA-Mφ-EVs, or SEA-Mφ-EVs with miR-33-knockdown via the tail-vein. qRT-PCR (C-G) and immunoblot (H) analysis of fibrotic markers (α-SMA, Col-I, and Col-III) in HSCs. Representative images of Masson’s trichrome staining showed collagen deposition (blue staining) in the liver (I). Scale bar, 200 μm. The percentage of the fibrotic area was quantified from the stained liver sections (J). Analysis of the whole liver collagen content was performed with a hydroxyproline assay (K). Graphical abstract of the mechanism by which *S*. *japonicum* infection/SEA utilizes Mφ-derived EVs to promote HSC activation and liver fibrosis (L). All graph data are expressed as the mean ± SD of 4 mice per group and are representative of two independent experiments. *P < 0.05, **P < 0.01, ***P<0.001. Created with BioRender.com.

Finally, we validated the role of SEA-Mφ-EVs in HSC activation and liver fibrosis in *S*. *japonicum*-infected mice. Similar to *in vitro* SEA-stimulated Mφ, primary hepatic Mφ from *S*. *japonicum*-infected mice also showed an increased expression of miR-33 compared with that from normal mice (Fig 8A). Likewise, EVs derived from Mφ of *S*. *japonicum*-infected mice also contained a higher abundance of miR-33 (Fig 8A). The experimental design is shown in Fig 8B. Mφ-EVs or SEA-Mφ-EVs with or without miR-33-knockdown were injected into *S*. *japonicum*-infected mice via the tail vein. qRT-PCR and immunoblot analysis revealed that HSCs from *S*. *japonicum*-infected mice displayed decreased SOCS3 expression and pronounced activation with increased levels of autocrine TGF-β1 and fibrotic markers (α-SMA, Col-I, and Col-III) compared to HSCs from uninfected mice (Fig 8C–8H). Consistent with the *in vitro* results, SEA-Mφ-EVs injection further decreased SOCS3 expression and increased the levels of TGF-β1 and fibrotic markers (α-SMA, Col-I, and Col-III) in HSCs of *S*. *japonicum*-infected mice, whereas SEA-Mφ-EVs with miR-33-knockdown had none of these effects (Fig 8C–8H). Furthermore, both Masson trichrome staining and hydroxyproline assay showed exacerbated liver fibrosis in *S*. *japonicum*-infected mice treated with SEA-Mφ-EVs, but not SEA-Mφ-EVs with miR-33-knockdown, as compared to Mφ-EVs-treated controls (Fig 8I–8K). In addition, no significant differences in liver egg burden were observed across groups (S5 Fig), suggesting SEA-Mφ-EVs inoculation with or without miR-33-knockdown does not affect infection burden. These results collectively demonstrate that SEA-Mφ-EVs utilize enclosed miR-33 to promote HSC activation and liver fibrosis in *S*. *japonicum*-infected mice.

Taken together, as shown in Fig 8L, during *S*. *japonicum* infection SEA induces Mφ to secrete abundant miR-33-enriched EVs, which are transferred to enhance autocrine TGF-β1 production by targeting SOCS3 in HSCs, thereby promoting HSC activation and liver fibrosis.

## Discussion

The primary pathological change of hepatic schistosomiasis is egg-induced granuloma and liver fibrosis which is inextricably linked to morbidity and mortality [1]. HSC activation plays an essential role in the pathogenesis of liver fibrosis [6,7]. As an important component of the egg granuloma, hepatic Mφ produce various cytokines and chemokines that initiate and/or perpetuate HSC activation through paracrine manners [5,10]. The current study demonstrated that upon SEA stimulation Mφ secrete abundant EVs carrying miR-33 to promote HSC activation by targeting SOCS3 expression and activating autocrine TGF-β1 signaling in HSCs, thereby accelerating the progression of hepatic fibrosis.

Accumulating evidence shows that EVs have been considered as a novel mode of cell-cell communications for delivering pro-fibrotic and/or anti-fibrotic signals during the fibrotic process in the liver, lung, kidney, etc [30]. Upon stimulations, Mφ can release EVs carrying different molecular components to regulate fibrogenesis depending on the microenvironment [15]. Herein, we found that SEA-stimulated Mφ or hepatic Mφ from *S*. *japonicum*-infected mice released miR-33-rich EVs to HSC activation and liver fibrosis. Similarly, LPS stimulation has been shown to induce miR-103-3p-enclosed EVs to target HSC and promote its activation and liver fibrosis [31]. Besides, Wang et al. demonstrated that silica can stimulate Mφ to produce EVs carrying miR-125a-5p and facilitate the transdifferentiation of fibroblasts into myofibroblasts, thereby promoting pulmonary fibrosis in silicosis [32]. High glucose has been shown to stimulate Mφ to secrete EVs containing TGF-β1 mRNA that promotes the activation of glomerular mesangial cells and ultimately leads to kidney fibrosis [33]. Therefore, in addition to paracrine soluble signaling molecules [7], paracrine EVs by local Mφ are currently considered as an important mechanism for delivering molecular information to regulate the fibrotic process under pathological conditions.

TGF-β binds and phosphorylates the type I receptor to activate HSCs [7]. Schistosome infection or SEA stimulation can induce Mφ to secrete TGF-β1 for activating HSCs [34,35]. We found that TGF-β type I receptor inhibitor almost completely abolished the ability of SEA-Mφ-EVs to activate HSCs. Mechanically, SEA-Mφ-EVs did not directly transfer TGF-β1 protein or mRNA but stimulated autocrine TGF-β1 production in the recipient HSCs. Hence, our data demonstrate, for the first time, SEA stimulation *in vitro* can indirectly induce TGF-β1 production in HSCs by utilizing SEA-Mφ-derived EVs to promote HSC activation. In addition, although we also demonstrated that *in vivo* treatment of SEA-Mφ-derived EVs further increased TGF-β1 expression and promoted HSC activation and hepatic fibrosis in *S*. *japonicum*-infected mice, the degree of this contribution to HSC activation and liver fibrosis in *S*. *japonicum*-infected mice remains to be determined.

A variety of miRNAs are identified as profibrogenic or antifibrotic factors that can regulate the process of HSC activation and hepatic fibrosis by targeting fibrosis-associated signaling pathways, such as TGF-β1/Smad, Wnt/β-catenin, Hedgehog, etc [36]. As vehicles, EVs act as important intercellular shuttles to deliver molecular biological signals [24]. However, it remains largely unclear what specific EVs within local Mφ contribute to the miRNA profile of HSCs in the hepatic fibrosis process remains largely unclear. Up to now, only a few *in vitro* studies regarding the alteration of the miRNA profile in HSCs by Mφ-derived EVs. For instance, upon LPS stimulation, THP-1 macrophages can secret exosomal miR-103-3p to promote HSC activation by targeting Krüpple-like factor 4 [31]. We currently demonstrate that both *in vitro* SEA-stimulated Mφ and primary hepatic Mφ from *S*. *japonicum*-infected mice secrete miR-33-enriched EVs to alter the miRNA profile in HSCs. Thus, our newly identified exosomal miRNA may provide a new target for the therapy of hepatic fibrosis in future. In addition, increased levels of miR-33 in Mφ were also observed in other inflammatory fibrotic diseases such as kidney fibrosis and lung fibrosis [37,38]. Therefore, we are prone to speculate that SEA may not be specifically and directly responsible for increased miR-33 in Mφ, which is more likely related to the inflammatory process induced by SEA.

SOCS3 has been reported to negatively regulate TGF-β1 production and liver fibrosis in hepatocellular carcinoma or hepatitis C virus (HCV) infection [27,39]. Here, we observed decreased SOCS3 expression in both SEA-Mφ-EVs-stimulated HSCs and primary HSCs from *S*. *japonicum*-infected mice treated with SEA-Mφ-EVs. Furthermore, miR-33 was for the first time identified to be enriched in Mφ-derived EVs for targeting SOCS3 in HSCs during schistosome infection. Similarly, hepatocyte-derived EVs have also been reported to target SOCS3 by transferring miR-19a to HSCs during HCV infection [39]. Therefore, as can be seen in different liver diseases, EVs with different cellular sources and contents in the liver can achieve the common modulation of the critical gene for HSC activation, which advances the current understanding of hepatic fibrosis.

Our study also has two main limitations that should be addressed in future study. In addition to miR-33, other miRNAs or biological molecules are also likely to exist for SEA-Mφ-EVs-mediated HSC activation. The other limitation is that the relative contribution of miR-33 to this process in *S*. *japonicum*-infected mice remains uncertain.

Overall, we demonstrate that *S*. *japonicum* infection or SEA stimulates hepatic Mφ to secrete abundant EVs, which are enriched with miR-33 and transferred to neighboring HSCs to enhance their autocrine TGF-β1 production and activation by targeting SOCS3 and ultimately promote liver fibrosis. Our findings unravel a previously unrecognized Mφ-EVs-based target for preventing liver fibrosis in hepatic schistosomiasis.

## Supporting information

S1 Fig Transmission electron microscopy identification of EVs. EVs were enriched from SEA (320 μg) and the supernatants of SEA-stimulated Mφ (cultured in 8 ml DMEM containing 10% EV-depleted FBS and 40 μg/ml SEA) by using centrifugation and then identified with a transmission electron microscope. Scale bar, 100 nm.
(TIF)

S2 Fig Nanoparticle tracking analysis of Mφ-EVs and SEA-Mφ-EVs. Nanoparticle tracking analysis (NTA) of Mφ-EVs and SEA-Mφ-EVs was performed to determine vesicle size distributions.
(TIF)

S3 Fig Immunoblot and qRT-PCR analysis of TGF-β1 levels in Mφ-EVs and SEA-Mφ-EVs. GAPDH was used as a protein loading control.
(TIF)

S4 Fig Agarose gel electrophoresis of SEA-Mφ-EVs treated with or without RNase A and Triton X-100. (TIF)

S5 Fig Schistosome egg burden in mouse liver. Animal experimental design is shown in Fig 8B. *S*. *japonicum*-infected mice were injected with Mφ-EVs, SEA-Mφ-EVs, or SEA-Mφ-EVs with miR-33-knockdown via the tail-vein. The number of eggs extracted from the livers of mice was determined by microscopic examination.
(TIF)

S1 Table Sequences of RT-PCR primers. (DOCX)

S2 Table The concentrations for western blot antibodies. (DOCX)

S1 Data Excel spreadsheet containing, in separate sheets, the underlying numerical data and statistical analysis for Figs 1B, 1C, 1E, 1F, 2B, 3B, 3C, 4B, 4C, 4E, 4F, 5A, 5B, 5C, 6A, 6D, 6E, 6H, 6I, 6J, 6K, 6N, 7B, 7C, 7D, 7E, 8A, 8C, 8D, 8E, 8F, 8G, 8I, 8K, S2B, S3A, S3B, and S5. (XLSX)
