# Supplementary figures and images for: Schistosome egg antigen stimulates the secretion of miR-33-carrying extracellular vesicles from macrophages to promote hepatic stellate cell activation and liver fibrosis in schistosomiasis
Source: PLoS Negl Trop Dis. 2023 May 30;17(5):e0011385. doi: 10.1371/journal.pntd.0011385 (PMC10256196; doi:10.1371/journal.pntd.0011385)

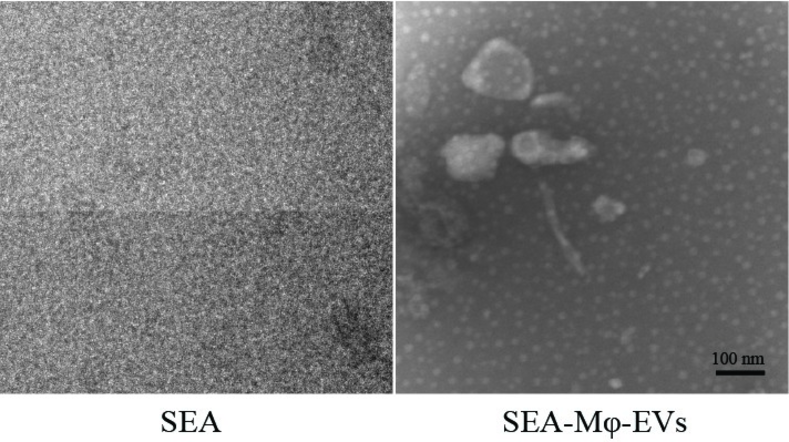

Supplement: S1 Fig — EVs were enriched from SEA (320 μg) and the supernatants of SEA-stimulated Mφ (cultured in 8 ml DMEM containing 10% EV-depleted FBS and 40 μg/ml SEA) by using centrifugation and then identified with a transmission electron microscope. Scale bar, 100 nm. (TIF) [file pntd.0011385.s001.tif]

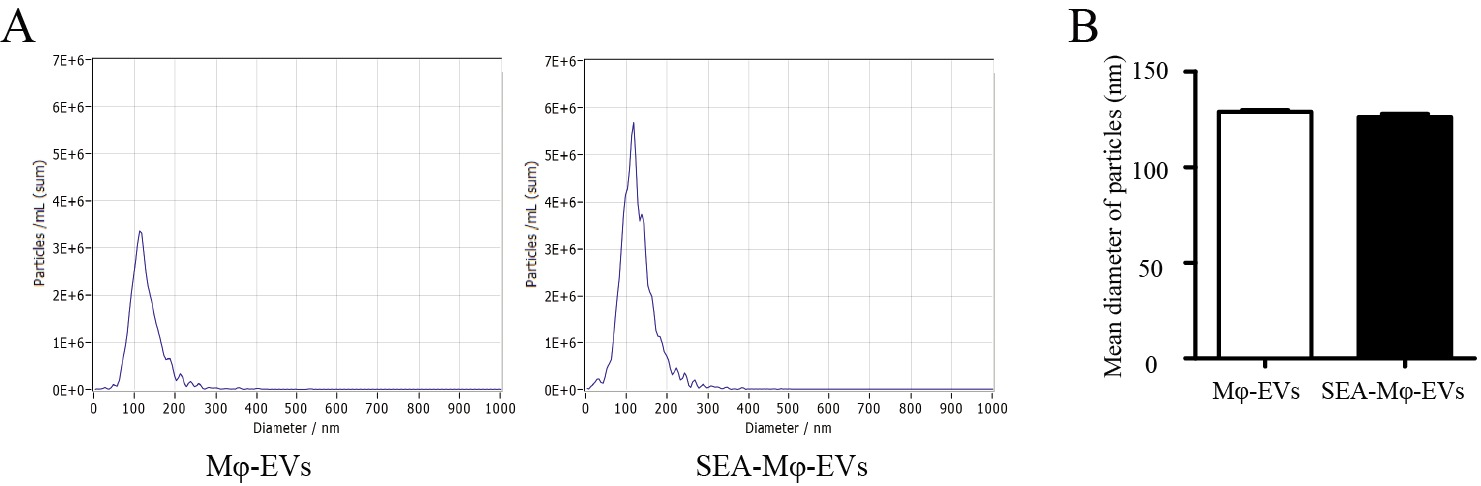

Supplement: S2 Fig — Nanoparticle tracking analysis (NTA) of Mφ-EVs and SEA-Mφ-EVs was performed to determine vesicle size distributions. (TIF) [file pntd.0011385.s002.tif]

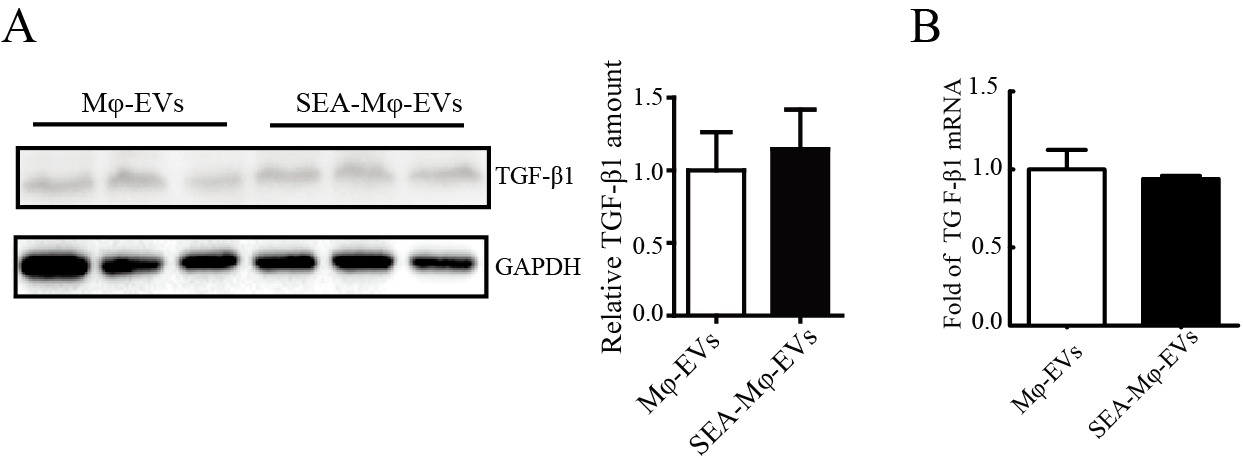

Supplement: S3 Fig — GAPDH was used as a protein loading control. (TIF) [file pntd.0011385.s003.tif]

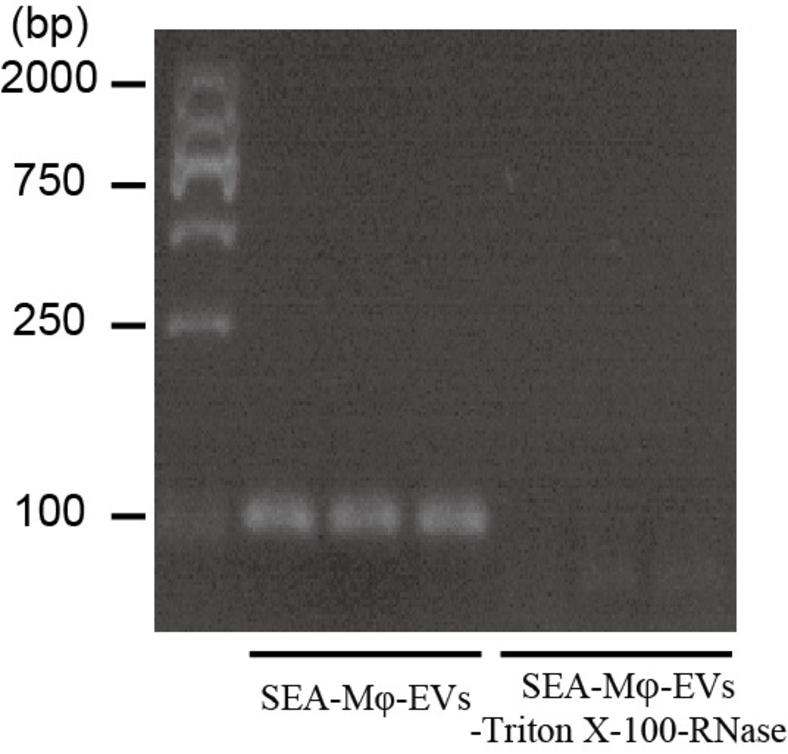

Supplement: S4 Fig — (TIF) [file pntd.0011385.s004.tif]

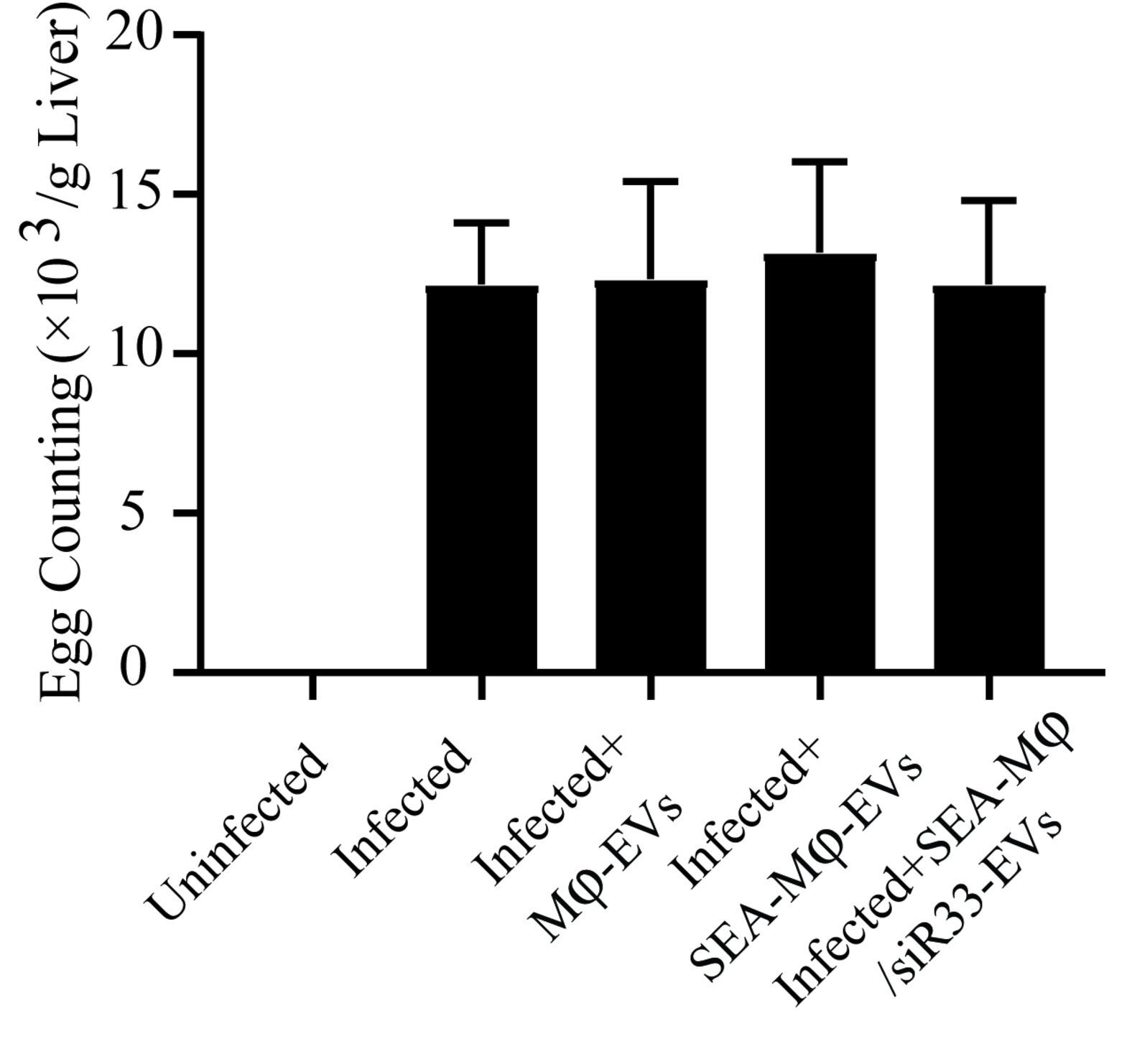

Supplement: S5 Fig — Animal experimental design is shown in Fig 8B. S. japonicum-infected mice were injected with Mφ-EVs, SEA-Mφ-EVs, or SEA-Mφ-EVs with miR-33-knockdown via the tail-vein. The number of eggs extracted from the livers of mice was determined by microscopic examination. (TIF) [file pntd.0011385.s005.tif]
