# Supplementary material for: Schistosome egg antigen stimulates the secretion of miR-33-carrying extracellular vesicles from macrophages to promote hepatic stellate cell activation and liver fibrosis in schistosomiasis
Source: PLoS Negl Trop Dis. 2023 May 30;17(5):e0011385. doi: 10.1371/journal.pntd.0011385 (PMC10256196; doi:10.1371/journal.pntd.0011385)
Supplement: S1 Table — (DOCX) [file pntd.0011385.s006.docx]

**Supplementary S1 Table. Sequences of RT-PCR primers.**

| **Gene** | **Primers (5’ to 3’)** | |
| --- | --- | --- |
| α-SMA | Forward | CCGCCATGTATGTGGCTATT |
|  | Reverse | CAGTTGTACGTCCAGAGGCATA |
| Col-I | Forward | GCTCCTCTTAGGGGCCACT |
|  | Reverse | CCACGTCTCACCATTGGGG |
| Col-III | Forward | ACGTAGATGAATTGGGATGCAG |
|  | Reverse | GGGTTGGGGCAGTCTAGTG |
| SOCS3 | Forward | ATGGTCACCCACAGCAAGTTT |
|  | Reverse | TCCAGTAGAATCCGCTCTCCT |
| TGF-β1 | Forward | TGACGTCACTGGAGTTGTACGG |
|  | Reverse | GGTTCATGTCATGGATGGTGC |
| β-actin | Forward | GGCTGTATTCCCCTCCATCG |
|  | Reverse | CCAGTTGGTAACAATGCCATGT |
| miR-33 | Forward | AGCCTGCTGGGCTCTCTGAGAC |
|  | Reverse | AGCCGCTACCATGACATTCCAG |
| miR-148b | Forward | TCAGTGCATCACAGAACTTTGT |
|  | Reverse | GCGAGCACAGAATTAATACGAC |
| U48 | Forward | TGACCCCAGGTAACTCTGAGTGTGT |
|  | Reverse | AACTCAAGGTTCTTCCAGTCACG |
