# Supplementary material for: Schistosome egg antigen stimulates the secretion of miR-33-carrying extracellular vesicles from macrophages to promote hepatic stellate cell activation and liver fibrosis in schistosomiasis
Source: PLoS Negl Trop Dis. 2023 May 30;17(5):e0011385. doi: 10.1371/journal.pntd.0011385 (PMC10256196; doi:10.1371/journal.pntd.0011385)
Supplement: S2 Table — (DOCX) [file pntd.0011385.s007.docx]

**Supplementary S2 Table. The concentrations for western blot antibodies.**

| **Antibody** | **Dilution** |
| --- | --- |
| TGF-β1 | 1:1000 |
| Collagen I | 1:1000 |
| Collagen III | 1:1000 |
| α-SMA | 1:1000 |
| SOCS3 | 1:1000 |
| SMAD3 | 1:1000 |
| p-SMAD3 | 1:1000 |
| CD63 | 1:2000 |
| Calreticulin | 1:2000 |
| TSG101 | 1:1000 |
| GAPDH | 1:1000 |
| β-ACTIN | 1:1000 |
| HRP-goat anti-rabbit IgG | 1:5000 |
| HRP-rabbit anti-mouse IgG | 1:2000 |
